# Multi‐isocentric 4*π* volumetric‐modulated arc therapy approach for head and neck cancer

**DOI:** 10.1002/acm2.12164

**Published:** 2017-08-20

**Authors:** Vallinayagam Shanmuga Subramanian, Vellaiyan Subramani, Srinivas Chilukuri, Murugesan Kathirvel, Gandhi Arun, Shanmugam Thirumalai Swamy, Kala Subramanian, Antonella Fogliata, Luca Cozzi

**Affiliations:** ^1^ Yashoda Super Specialty hospital Department of Radiation Oncology Hyderabad India; ^2^ Research and Development Centre Bharathiar University Coimbatore India; ^3^ Department of Radiation Oncology All India Institute of Medical Sciences New Delhi India; ^4^ Radiotherapy and Radiosurgery Humanitas Cancer Center and Research Hospital Milan Italy; ^5^ Department of Biomedical Sciences Humanitas University Milan Italy

**Keywords:** 4*π*, head and neck cancer, RapidArc, VMAT

## Abstract

**Objectives:**

To explore the feasibility of multi‐isocentric 4*π* volumetric‐modulated arc therapy (MI4*π*‐VMAT) for the complex targets of head and neck cancers.

**Methods:**

Twenty‐five previously treated patients of HNC underwent re‐planning to improve the dose distributions with either coplanar VMAT technique (CP‐VMAT) or noncoplanar MI4*π*‐VMAT plans. The latter, involving 3–6 noncoplanar arcs and 2–3 isocenters were re‐optimized using the same priorities and objectives. Dosimetric comparison on standard metrics from dose‐volume histograms was performed to appraise relative merits of the two techniques. Pretreatment quality assurance was performed with IMRT phantoms to assess deliverability and accuracy of the MI4*π*‐VMAT plans. The gamma agreement index (GAI) analysis with criteria of 3 mm distance to agreement (DTA) and 3% dose difference (DD) was applied.

**Results:**

CP‐VMAT and MI4*π*‐VMAT plans achieved the same degree of coverage for all target volumes related to near‐to‐minimum and near‐to‐maximum doses. MI4*π*‐VΜΑΤ plans resulted in an improved sparing of organs at risk. The average mean dose reduction to the parotids, larynx, oral cavity, and pharyngeal muscles were 3 Gy, 4 Gy, 5 Gy, and 4.3 Gy, respectively. The average maximum dose reduction to the brain stem, spinal cord, and oral cavity was 6.0 Gy, 3.8 Gy, and 2.4 Gy. Pretreatment QA results showed that plans can be reliably delivered with mean gamma agreement index of 97.0 ± 1.1%.

**Conclusions:**

MI4*π*‐VMAT plans allowed to decrease the dose‐volume‐metrics for relevant OAR and results are reliable from a dosimetric standpoint. Early clinical experience has begun and future studies will report treatment outcome.

## 1 INTRODUCTION

IMRT for head and neck cancer (HNC) has been the standard practice for the last decade as it has shown to reduce xerostomia and improve associated quality of life (although such improvement did not resulted statistically significant).^1^ Since its introduction in 2008, volumetric‐modulated arc therapy (VMAT) has been extensively evaluated for treating HNC.^2^, ^3^, ^4^, ^5^, ^6^, ^7^, ^8^ The literature suggests that both treatment efficiency and sparing of organs at risk (OAR) are superior with VMAT compared to conventional static field IMRT (SF‐IMRT), although questioned by some authors.^2^, ^3^ Early clinical outcome reports showed comparable toxicity and local control with respect to IMRT.^4^, ^5^, ^6^ Advanced planning methods, like knowledge‐based automated planning strategies have also been explored to further improve the level of OAR sparing and the harmonization of the results at an interpatient and an interplanner level.^9^

Nevertheless, due to the anatomical complexity and several trade‐offs between target coverage and OAR sparing, the use of a simple coplanar approach to the arc geometry setting being currently used seems to leave space for improvement. More recently, some groups explored the possibility to deliver SF‐IMRT with conventional c‐arm linear accelerators using most of the 4*π* space, i.e., making extensive use of noncoplanar beam arrangements and creating complex delivery trajectories for the couch‐gantry‐collimator system around the patient.^10^, ^11^, ^12^, ^13^, ^14^, ^15^ These investigators focused on stereotactic irradiation in the brain, lungs, and prostate and have shown that significantly sharper dose gradients can be achieved with this approach. These studies concluded that the 4*π* technique reduced mean or maximum doses to all OAR and may allow for safe dose escalation. The original investigations published provided evidence of benefit and proof of principle for smaller tumors. A study involving the 4*π* approach to SF‐IMRT for HNC was also attempted but it was for small and recurrent cancers.^16^ The question of applicability of 4*π* techniques to truly large target volumes and its feasibility for conventional IMRT/VMAT treatments remains unaddressed for HNC. The aim of this study was to explore multi‐isocentric 4*π* volumetric‐modulated arc therapy (MI4*π*‐VMAT) plans in terms of dosimetry and delivery and comparing it with best coplanar VMAT(CP‐VMAT) plans for the irradiation of HNC patients characterized by large targets and the presence of several organs at risk. Deliverability was addressed in terms of dosimetric accuracy. In the absence of an automated collision avoidance engine, this aspect was qualitatively addressed with the pretreatment quality assurance procedures performed with a body phantom.

## 2 MATERIALS AND METHODS

Institutional scientific and ethics board approved this study. Twenty‐five previously treated HNC patients with two coplanar volumetric arcs (CP‐VMAT) were included in a retrospective preclinical planning study. Patient characteristics are summarized in Table 1. For each patient, the gross tumor volume (GTV) was defined as the macroscopic tumor seen on imaging, while the clinical tumor volumes (CTVs) were defined as per standard institutional practice for HNC. CTV nodal volumes were defined as per standard RTOG protocol.^17^ Planning target volume (PTV) was generated by isotropic expansion of CTV by 0.5 cm. Each PTV was defined as the mutual subtraction of each other, so they were not mutually including each other. For all the patients, the following organs at risk were defined: parotids, oral cavity, esophagus, trachea, larynx, pharyngeal muscles, mandible, temporomandibular joint, middle ear, spinal cord, and brain stem. For the spinal cord, the near‐to‐maximum dose constraint was set to 45 Gy to 1% of its volume (50 Gy for the brain stem). For the parotids, the mean dose was aimed to be lower than 32 Gy. For the other structures, the planning strategy was to minimize as much as reasonably possible their involvement. Standard dose prescription was used (PTV‐high: 70 Gy, PTV‐mid: 60/63 Gy and PTV‐low: 56 Gy).

**Table 1:** Patients characteristics

Patient characteristics	Number of patients/value
Median age	56 yr
Age < 65 yr	15
Age > 65 yr	10
Sex	
Male	14
Female	11
T Stage	
T1	0
T2	4
T3	12
T4	9
N Stage	
N0	4
N1	6
N2	14
N3	1
Location	
Nasopharynx	4
Oropharynx	6
Oral cavity	8
Larynx	7
PTV volumes	
PTV‐high	
Range	36–168 cm^3^
Median	94 cm^3^
PTV‐mid	
Range	169–898 cm^3^
Median	492 cm^3^
PTV‐low	
Range	496–1162 cm^3^
Median	720 cm^3^

Treatment planning was performed for 6 MV beams on a Clinac‐iX linear accelerator (Varian Medical Systems, Palo Alto, USA) equipped with a 120 leaves Millennium Multileaf collimator. Inverse planning and dose calculations were performed using the Progressive resolution optimization algorithm (PRO) and Acuros XB dose calculation engine in the Eclipse treatment planning system (version 13.1, Varian Medical Systems, Palo Alto, USA). Dose calculation was performed on a 2.5 mm matrix on planning CT datasets acquired with 3 mm slice thickness. CP‐VMAT plans were optimized with two full‐arcs (720 degrees) with collimator angles in the range of 10–20° according to the patients anatomy. The CP‐VMAT plans were re‐optimized starting from the clinically accepted ones to improve the reference dose distributions. Aims were to achieve the highest possible dose conformity to the target with the least involvement of the organs at risk. Multiple planners calculated the CP‐VMAT plans but the selection of the final plan was made on a shared consensus. The optimal plans were selected in terms of numerical plan quality metrics, these were the ones with the “best” results for each of the planning dose‐volume objectives. No knowledge‐based planning tools were applied since not available at the clinic.

**Figure 1:**
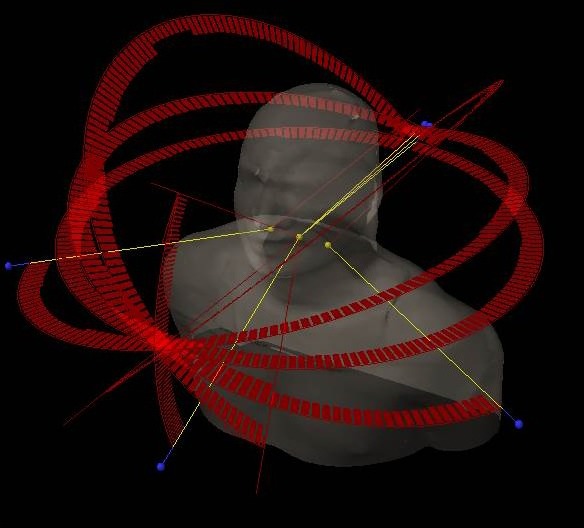
The typical field geometry for an example case.

MI4*π*‐VMAT plans were optimized using the same objectives and priorities as the co‐planar ones. Plan geometry consisted of 3–6 arcs with 2–3 isocenters which were manually selected to avoid any risk of collisions (and verified qualitatively during the pretreatment dosimetric verification with the body phantom) which might occur during the noncoplanar arc trajectory. Extreme care was taken to assign isocenters such that there was a minimum of 10 cm clearance between the patient and gantry‐collimator system as well as between the collimator and couch surfaces to avoid risks of collisions. For each patient, MI4*π*‐VMAT plan geometry was validated for delivery by simulating the planned field geometry with their immobilization system and actual isocenters at place. This simulation also ruled out the possibility of collision during noncoplanar arc trajectories. The typical field geometry for an example case is illustrated in Fig. 1. The average total arc length for twenty‐five MI4*π*‐VMAT plans was 1115 ± 228 degrees with a maximum of 1358 degrees. All the plans had one full coplanar arc in addition to the noncoplanar arcs. Typical field geometry consisted of one full‐arc with couch angle 0 degree, two partial arcs (arc length of ± 210°) with average couch rotation of ± 45°, and two more partial arcs (arc length of ± 250°) with couch rotation of ± 15°. The arc selection in general was determined according to the following strategy: when the overlap between PTV and the parotid glands (or other relevant structures in addition) was smaller than 20% of the glands, then three arcs were selected (one coplanar and two noncoplanar with average couch rotation of ± 45°). In the other cases, two additional arcs (with couch rotation of ± 15°) were added. For a few patients with intracranial extension, one more arc (60° arc length) with a couch rotation of 90° was added.

For all plans, fixed jaws setting was applied with the restriction of a maximum x‐field size smaller than 15–16 cm to prevent loss in modulation power induced by insufficient over‐travel movement of the MLC leaves in that direction.

All plans were optimized by the same experienced senior planner, blind to the outcome of the CP‐VMAT plans. The plan optimization was performed interactively without definite and absolute rules about the number of objectives per OAR. All objectives were simultaneously enforced (i.e., targets and OAR at the same time from the start). The final plans were obtained with trial and error process during the optimization. The plans selected for the comparison, as for the clinical CP‐VMAT plans, were those which “maximized” their adherence to the clinical objectives. The optimization objectives were defined in the same way for the two groups of plans. The position of these objectives was individually defined during the interactive optimization of the CP dataset. No absolute rules were applied concerning the distance between the objectives and the DVH line but rather the optimization was pushed toward the best results per each structure.

Dose‐volume histograms (DVHs) were calculated for the PTV and OAR. Since there was minor variation in PTV dose prescription among patients, all the target volumes were scored in terms of percentage dose rather than absolute dose. The DVH was assessed quantitatively using a number of appropriate metrics, which included the mean dose (D_mean_), the dose received by 1% of the PTV/OAR volume (D_1%_), D_98%_, D_95%,_ D_5%–95%_, as well as a variety of V_xGy_ values. For each analyzed parameter, the mean values ±SD for the 25patients cohort were analyzed. The Wilcoxon matched‐paired signed‐rank test was applied to evaluate the level of significance of the observed difference between the dose‐volume metrics. The threshold for statistical significance was set at < 0.05.

The deliverability of the MI4*π*‐VMAT plans was tested by means of point and planar pretreatment dosimetry using the I'mRT phantom (IBA dosimetry, GmbH, Germany). Point dosimetry was carried out with the compact cylindrical ionization chamber CC13 (IBA dosimetry, GmbH, Germany) ion chamber with a 0.13 cm^3^ active volume (cross‐calibrated against a secondary standard 0.65 cm^3^ Farmer type chamber) and the percentage variation was calculated between predicted doses from Eclipse and measured dose in the I'mRT. Planar dosimetry was carried out using 2D array Matrixx^Evolution^ (which consists of 1020 parallel plate ion chambers (32 × 32 matrix) arranged in an active area of 24.4 × 24.4 cm^2^ with a 7.62 mm center‐to‐center distance between chambers) in a multicube phantom (IBA dosimetry, GmbH, Germany) without resetting couch, gantry and collimator and isocenters. Omnipro IMRT QA software (IBA dosimetry, GmbH, Germany) was used to perform global gamma agreement index (GAI) analysis with criteria of 3 mm distance to agreement (DTA) and 3% dose difference (DD). GAI was used to quantify the agreement between the predicted and measured dose distribution at the isocenter plane.

## 3 RESULTS

**Figure 2:**
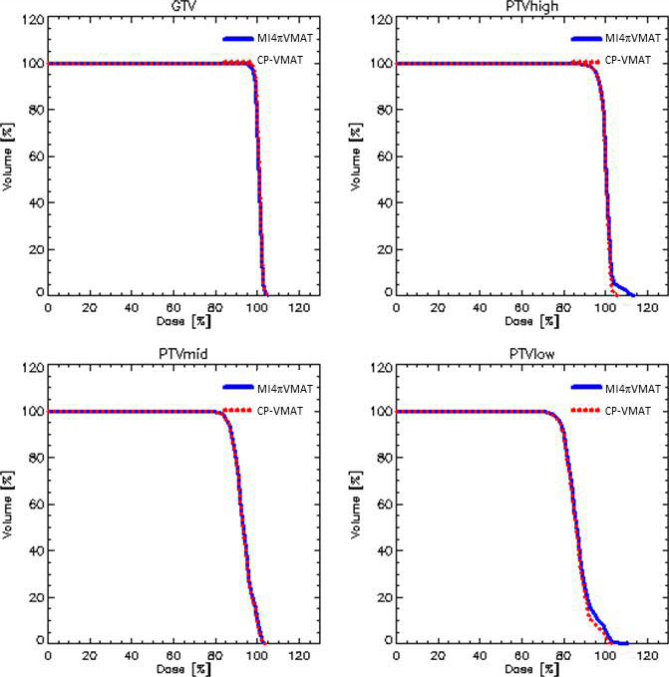
Average dose‐volume histograms for the target volumes.

**Figure 3:**
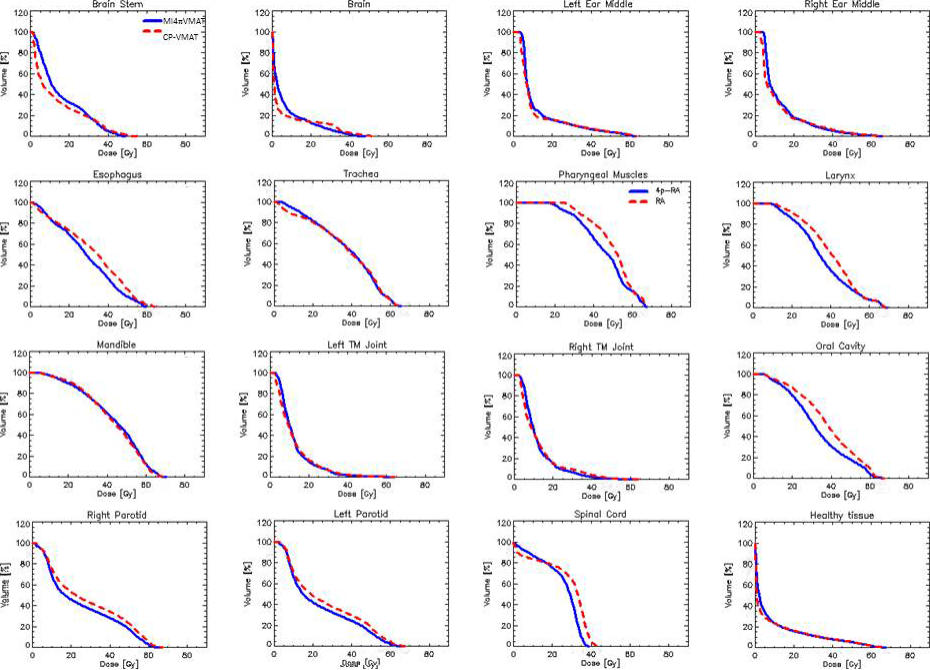
Average dose‐volume histograms for the organs at risk.

**Figure 4:**
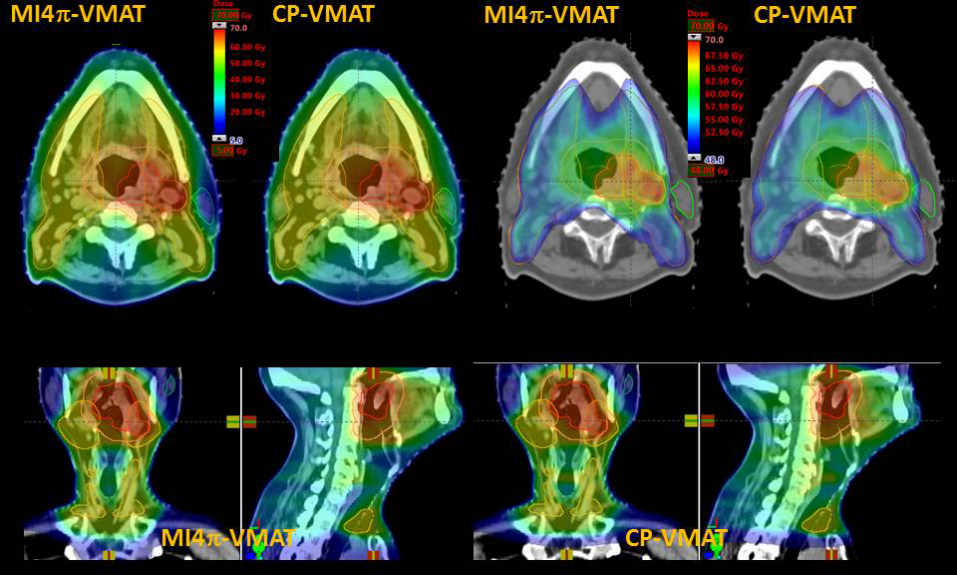
The typical dose distributions in axial, coronal, and sagittal planes for one patient and the two techniques. The color‐wash display is set to 5–70 Gy to display the low dose bath and to 48–70 Gy to display the dose conformality.

Figures 2 and 3 show the DVH parameters for PTV and OAR depicting the best CP‐VMAT and MI4*π*‐VMAT plan comparison. Figure 4 shows the typical dose distributions of both the techniques in axial, coronal, and sagittal planes for a patient. The color‐wash display is set to 5–70 Gy to display the dose bath and to 48–70 Gy to display dose conformality.

From the qualitative inspection of the DVHs, the target coverage was similar between the best CP‐VMAT and MI4*π*‐VMAT plans with respect to conformity, homogeneity, and percentage doses. However, when the OAR doses were compared, the MI4*π*‐VMAT plans delivered significantly less dose to various relevant OAR. There was significant reduction in average mean doses with MI4*π*‐VMAT plans with respect to bilateral parotids by 3 Gy, oral cavity by 5 Gy, pharyngeal constrictors by 4.3 Gy, larynx by 4 Gy, and upper esophagus by 3.3 Gy. There was also significant reduction in the average maximum doses to the brain stem that was reduced by 6.0 Gy, to the spinal cord by 3.7 Gy and to the oral cavity by 2.4 Gy. For the brain, there was a statistically significant increase in the mean and low dose involvement due to a somehow broader dose bath delivery for the not coplanar arcs. The dose bath is represented by the dose to the healthy tissue (conventionally defined as the body's volume in the CT minus the encompass of all target volumes). Tables 2 and 3 show the results of the quantitative analysis conducted on the DVH for the various parameters considered for PTV and the subset of OAR where remarkable differences were observed.

**Table 2:** Summary of the quantitative analysis of the dose‐volume histograms of the target volumes of the two techniques. The percentage values are all relative to the nominal dose prescription to PTV‐high

Parameter		MI4*π*‐VMAT	CP‐VMAT	*P*‐value
PTV‐high				
Mean	100%	100.0 ± 0.0	100.0 ± 0.0	ns
D_2%_	< 105%	103.8 ± 2.5	103.3 ± 0.9	ns
D_95%_	> 95%	96.1 ± 2.5	95.7 ± 2.0	ns
D_98%_	Maximize	93.8 ± 4.5	93.5 ± 4.5	ns
PTV‐mid				
Mean	86–90% (60–63 Gy)	93.5 ± 1.9	93.4 ± 2.1	ns
D_2%_	< 105%	101.2 ± 2.3	100.5 ± 2.3	ns
D_95%_	> 81% (of 70 Gy)	87.2 ± 2.8	87.4 ± 2.7	ns
D_98%_	Maximize	85.2 ± 3.6	85.4 ± 3.5	ns
PTV‐low				
Mean	80% (56 Gy)	87.0 ± 4.2	86.3 ± 3.2	ns
D_2%_	< 105%	97.1 ± 6.4	96.6 ± 5.5	ns
D_95%_	> 76% (of 70 Gy)	80.4 ± 4.4	79.9 ± 3.9	ns
D_98%_	Maximize	77.8 ± 4.8	77.7 ± 4.7	ns
GTV				
Mean	100%	100.8 ± 0.6	100.9 ± 0.5	ns
D_2%_	< 105%	103.1 ± 0.9	103.2 ± 0.8	ns
D_98%_	Maximize	95.2 ± 4.1	95.3 ± 4.0	ns

ns, not significant.

**Table 3:** Summary of the quantitative analysis of the dose‐volume histograms of organs at risk for the two techniques

Parameter	Objective	MI4*π*‐VMAT	CP‐VMAT	*P*‐value
Brain stem				
D_1%_ (Gy)	<50 Gy	33.9 ± 10.8	39.9 ± 11.8	0.004
Brain				
Mean (Gy)	–	7.5 ± 8.5	6.8 ± 7.4	0.02
V_10 Gy_ (%)	–	22.4 ± 30.4	18.9 ± 31.6	0.04
Esophagus				
Mean (Gy)	Minimize	28.9 ± 7.1	32.2 ± 7.4	< 0.001
D_1%_ (Gy)	Minimize	52.6 ± 5.5	53.0 ± 5.7	0.1
V_30 Gy_ (cm^3^)	Minimize	6.9 ± 3.5	8.5 ± 3.1	0.02
Larynx				
Mean (Gy)	Minimize	36.2 ± 11.3	40.3 ± 10.9	< 0.001
D_1%_ (Gy)	Minimize	58.2 ± 6.1	57.7 ± 6.4	0.3
Left parotid				
Mean (Gy)	<32 Gy	23.5 ± 6.7	26.3 ± 6.8	< 0.001
Right parotid				
Mean (Gy)	<32 Gy	25.2 ± 8.7	28.4 ± 9.2	< 0.001
Spinal cord				
D_1%_ (Gy)	<45 Gy	34.5 ± 2.7	38.2 ± 2.4	< 0.001
Left TM joint				
D_1%_ (Gy)	Minimize	22.7 ± 15.9	24.6 ± 17.3	0.1
Right TM joint				
D_1%_ (Gy)	Minimize	22.6 ± 18.0	24.3 ± 20.5	0.5
Oral cavity				
Mean (Gy)	Minimize	33.3 ± 9.5	38.3 ± 10.5	< 0.001
D_1%_ (Gy)	Minimize	56.9 ± 7.2	59.3 ± 5.6	0.02
Pharyngeal muscle				
Mean (Gy)	Minimize	45.6 ± 6.7	49.9 ± 7.5	< 0.001
D_1%_ (Gy)	Minimize	62.5 ± 4.9	62.3 ± 4.2	0.3
Healthy tissue				
Mean (Gy)	Minimize	9.4 ± 2.9	8.9 ± 2.7	< 0.001
V_10 Gy_ (%)	Minimize	23.8 ± 7.6	23.3 ± 7.3	ns

The average monitor units for MI4*π*‐VMAT and best CP‐VMAT plans were 525.4 ± 77.9 and 547.8 ± 70.2, respectively. MI4*π*‐VMAT plans exhibit lower average monitor units in comparison with the best CP‐VMAT plans although this difference was not statistically significant (*P* = 0.05). On the other hand, average total treatment time for MI4*π*‐VMAT plans (611.5 ± 76.6 s) was 3.65 times higher than that of best CP‐VMAT plans (167.3 ± 30.4, *P* = 0.0001).

**Figure 5:**
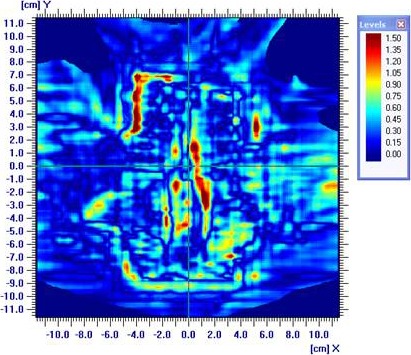
Typical gamma analysis for MI4*π*‐VMAT plan.

All the 25 MI4*π*‐VMAT plans were simulated for noncollisional delivery in the treatment room with patient immobilization system in place. The average point dosimetry absolute dose variation (calculated versus delivery) at all the isocenters was 0.05 ± 0.93% while the average GAI (< 1) was 97.04 ± 1.08%. Both values were found to be acceptable as per the institutional quality assurance protocol (Point dose variation < 3% and global GAI > 95% with 3 mm DTA/3%DD). Typical gamma analysis for MI4*π*‐VMAT plan was shown in Fig. 5.

## 4 DISCUSSION

There is a perception that current IMRT and VMAT techniques have hit a plateau with respect to physically achievable dose distributions. One of the techniques that challenge this perception is the 4*π* approach, which involves the use of multiple noncoplanar beams using robotic couch and gantry on modern C‐arm linear accelerators. The studies from the University of California (Los Angeles, UCLA) have elegantly described this technique and have demonstrated significant sparing of OAR and discussed the potential for dose escalation in patients of lung, liver, prostate, HNC, and brain tumors.^9^, ^10^, ^11^, ^12^, ^13^

But these studies have used static field IMRT (SF4*π*‐IMRT) in noncoplanar workspace with limited arc length compared to MI4*π*‐VMAT arc trajectories. Since VMAT has shown improved treatment efficiency with lesser monitor units compared to SF‐IMRT, we have attempted a multi‐isocentric noncoplanar VMAT using approximately 880–1350 degrees of freedom.^2^, ^3^ Our approach fundamentally differs from theirs in terms of use of volumetric arcs (instead of vvSF‐IMRT) in noncoplanar workspace using multiple isocenters creating multiple spheres of dose clouds. Although the study by Woods et al., have concluded that mono‐isocentric noncoplanar VMAT yields inferior dosimetric outcomes compared to SF4*π*‐IMRT, the average arc length used was only 553 degrees in their mono‐isocentric noncoplanar VMAT plans, compared to a total arc length of 1115 ± 228 degrees in our technique.^14^ The arc length was limited in their study because of the possibility of collision whereas in our study this problem was overcome with the use of multiple isocenters wherein couch translation yielded extra degrees of freedom. This gives the optimizer additional room to reduce the dose to OAR without loosing the PTV coverage even for larger and complex targets. In our opinion, doubling the arc length with multiple isocenter has significantly improved the quality of the plan.

Our MI4*π*‐VMAT plans showed uniformly superior sparing for studied OAR compared to best CP‐VMAT plans without compromising dose conformity to planning target volumes. Concerning organs at risk, significant sparing was observed for brain stem, esophagus, larynx, parotids, oral cavity, and pharyngeal muscles, either for the mean or the near‐to‐maximum doses with an obvious potential benefit in terms of reduced risk of normal tissue complication probability. Reduction in mean and/or maximum doses to structures such as parotids, oral cavity, pharyngeal muscles, and larynx may have discernible clinical impact in terms of late xerostomia and dysphagia/aspiration that define HNC radiotherapy. The use of not coplanar arc settings might induce some broader dose bath to the tissues as it was reported for the brain. The clinical relevance of this increased bath should be considered with care. These results achieved by MI4*π*‐VMAT plans for large PTV volumes confirm that dosimetric gain can be achieved irrespective of volume of PTV. GAI results also showed that MI4*π*‐VMAT plans were within the clinically acceptable limits and comparable with that of CP‐VMAT.

Compared to standard IMRT, the dosimetric quality of the SF4*π*‐IMRT approach, might be mitigated by the decreased treatment efficiency especially in the context of nonautomated couch and gantry movements (for delivery of 3–5 Gy per fraction, the average delivery time was 49 min with nonautomated delivery and 26 min for automated delivery).^18^ In SF4*π*‐IMRT technique, we expect an increase in number of monitor units with increase in the number of fields. On the contrary, MI4*π*‐VMAT plans with 5–6 arcs exhibit lower average monitor units compared with two full‐arc best CP‐VMAT plans although the difference was not statistically significant.

The mean increase in total treatment time with MI4*π*‐VMAT plans (with manual couch and gantry movements) compared with best CP‐VMAT plans was 444.24 ± 68.08 s (approximately 7.4 min). With the availability of automated gantry and couch movements, the treatment efficiency possibly could be further enhanced to an extent where this technique potentially can be used as a standard for HNC IMRT. The total beam on time with MI4*π*‐VMAT appears to be similar to that of conventional SF‐IMRT for HNC, which also ranges from 8–12 min.^3^, ^4^

There are certain limitations to this approach. The foremost is the use of trial and error method to select the number of isocenters and arc trajectories thereby possibly under‐utilizing the degrees of freedom available in noncoplanar workspace unlike the SF4*π*‐IMRT planning where it is algorithm driven.

Several major parameters were modified between the compared techniques. These included the number of isocenters, the number of arcs, their length, and the couch angles. It is clear that each of those can differently contribute to the improvements in the plan quality reported in the study. Nevertheless, the concept of 4*π* deliver implies the simultaneous change of these and the attempt to maximize the benefit of their mutual interplay. It would have been hard and beyond the scope of this feasibility study to systematically appraise the role of each of those. What would be needed to guarantee the maximization of the quality of the plans is an automated engine capable to inspect the entire multidimensional space to identify the ideal trajectories. It is reasonable to anticipate that treatment planning systems will be capable to perform this task in the medium future time scale.

The other limitation of this approach is the need to manually verify the planned trajectories for the possibility of collision for each patient. Lastly, patient set‐up verification with volumetric imaging (CBCT) which is extremely crucial for this technique because of multiple isocenters and a very sharp dose fall off, is not possible in noncoplanar workspace. Furthermore, since there are no perfect solutions, any table rotation or more in general, any movement during the treatment delivery, might be affected by uncertainties due to mechanical limits of the motors and might pile‐up with the set‐up errors. The state‐of‐the‐art modern linear accelerators (e.g., the TrueBeam system) might have sub‐mm accuracy in their rotational axes which could mitigate these risks.^19^ A dedicated study about the robustness of the plans against the table rotation errors is out of the scope of this investigation and should be considered as a follow‐up study. Depending on the equipment and staff, this can require amendments in the CTV‐PTV margin definitions or other mitigation strategies. However, simple planar MV imaging can be performed which could further decrease the treatment efficiency.

Another limitation of the study is the use of a global 3% 3 mm criterion for the gamma analysis, which might hide finer discrepancies. Nevertheless, the criteria applied are the standard used for clinical practice and are appropriate for the large volumes involved in the study.

## 5 CONCLUSION

The results from our study show the dosimetric performance and feasibility of MI4*π*‐VMAT for HNC. Compared with CP‐VMAT plans, all dose‐volume metrics of relevant OAR decreased significantly without altering dose conformity for relatively large and complex PTV volumes. These plans are clinically deliverable with acceptable quality assurance. The improvements in hardware and availability of MI4*π*‐VMAT optimization algorithm with automated delivery can further improve the quality of plans as well as enhance treatment efficiency and thereby making it possible for this technique to be adopted for routine day‐to‐day clinical practice. Early clinical experience has begun and future studies will aim to report treatment outcomes.

## **CONFLICT OF INTERESTS**

L. Cozzi acts as Scientific Advisor to Varian Medical Systems and is Clinical Research Scientist at Humanitas Cancer Center. All other co‐authors have no conflicts of interest.

## Supporting information


**Data S1.** Multi‐isocentric 4π volumetric‐modulated arc therapy approach for head and neck cancer.
