# Supplementary material for: Multi‐isocentric 4π volumetric‐modulated arc therapy approach for head and neck cancer
Source: J Appl Clin Med Phys. 2017 Aug 20;18(5):293–300. doi: 10.1002/acm2.12164 (PMC5874945; doi:10.1002/acm2.12164)
Supplement: Supplementary file 1 — Data S1. Multi‐isocentric 4π volumetric‐modulated arc therapy approach for head and neck cancer. [file ACM2-18-293-s001.docx]

**Multi isocentric** 4π **Volumetric modulated arc therapy approach for head and neck cancer**

**Complementary materials**

**Figure 1c**

**
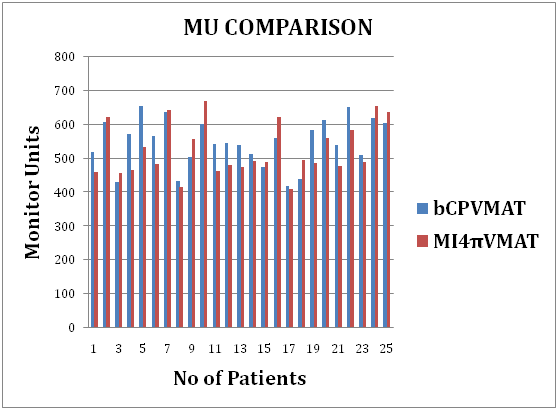
**

**Figure 2c**

**
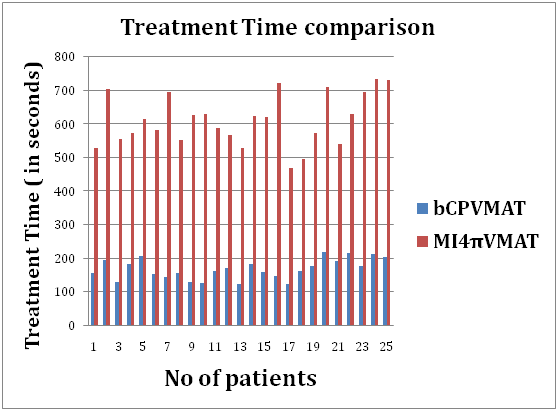
**

**Figure 3c**

**
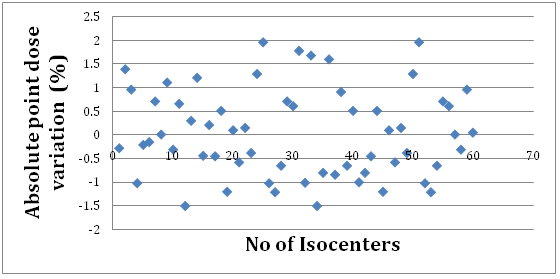
**

**Figure 4c**

**
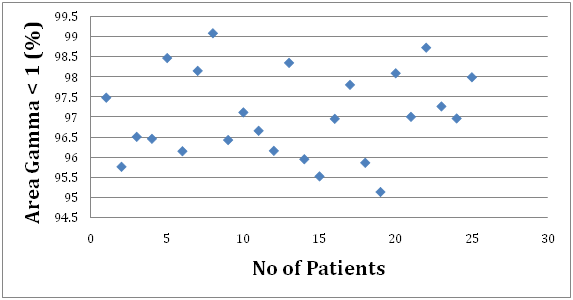
**

**Figure 5c**

**
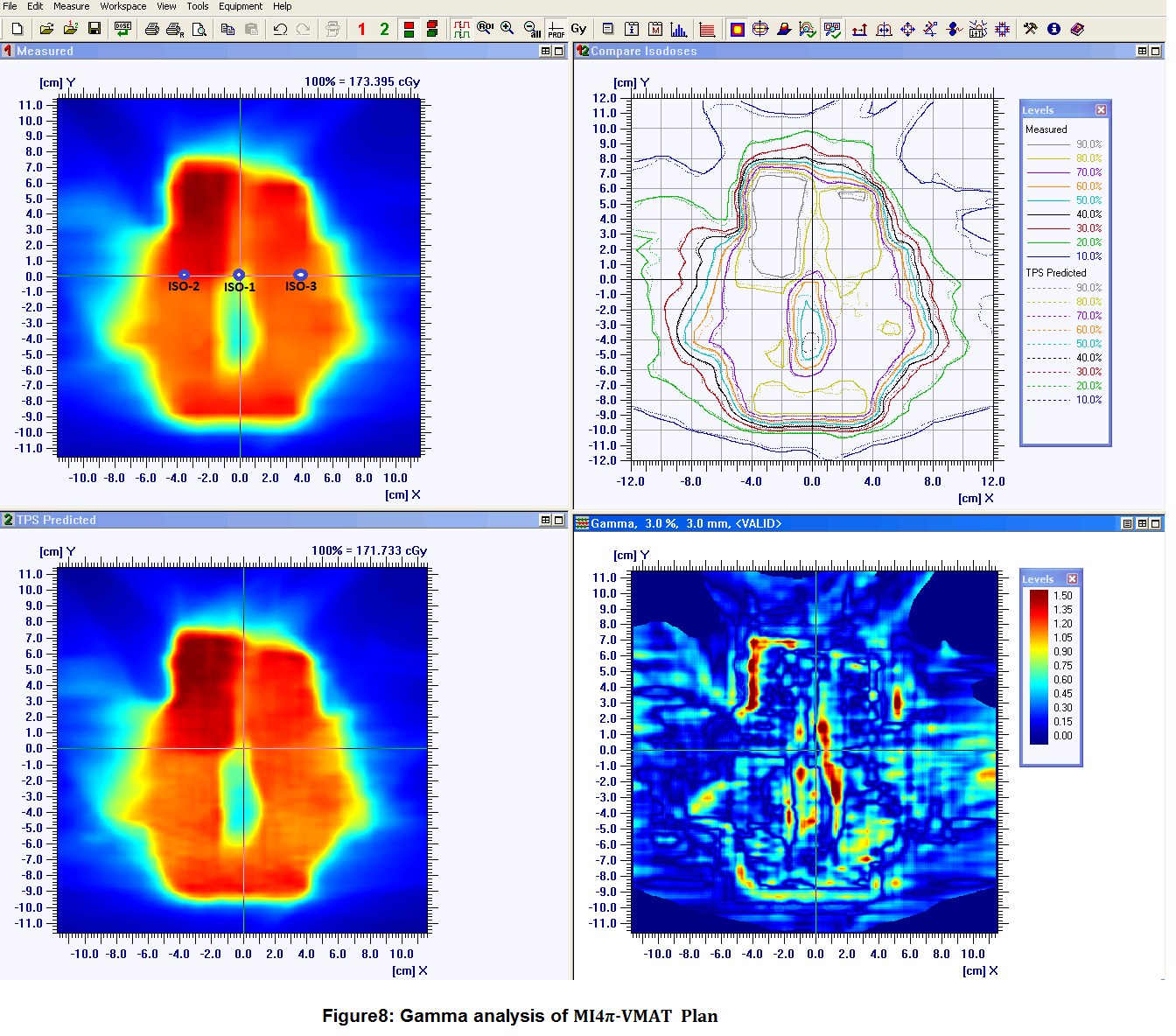
**
